# Akt1 and Akt2 Isoforms Play Distinct Roles in Regulating the Development of Inflammation and Fibrosis Associated with Alcoholic Liver Disease

**DOI:** 10.3390/cells8111337

**Published:** 2019-10-29

**Authors:** Karina Reyes-Gordillo, Ruchi Shah, Jaime Arellanes-Robledo, Ying Cheng, Joseph Ibrahim, Pamela L. Tuma

**Affiliations:** 1 Lipid Research Laboratory, VA Medical Center, Washington, DC 98100, USA; karrygor@gwu.edu (K.R.-G.); Ruchi.Shah@cshs.org (R.S.); jarellanes@gmail.com (J.A.-R.); yingcheng2004@gmail.com (Y.C.); bimoib@yahoo.com (J.I.); 2 Department of Biochemistry and Molecular Medicine, The George Washington University Medical Center, Washington, DC 20037, USA; 3 Department of Biology, The Catholic University of America, Washington, DC 20064, USA; 4 Laboratory of Hepatic Diseases; Catedras-CONACYT and National Institute of Genomic Medicine (INMEGEN), CDMX 64460, Mexico

**Keywords:** Akt1, Akt2, Akt3, Akt isoforms, alcoholic liver disease, liver fibrosis, inflammation, proliferation, migration

## Abstract

Akt kinase isoforms (Akt1, Akt2, and Akt3) have generally been thought to play overlapping roles in phosphoinositide 3-kinase (PI3K)-mediated-signaling. However, recent studies have suggested that they display isoform-specific roles in muscle and fat. To determine whether such isoform-specificity is observed with respect to alcoholic liver disease (ALD) progression, we examined the role of Akt1, Akt2, and Akt3 in hepatic inflammation, and pro-fibrogenic proliferation and migration using Kupffer cells, hepatic stellate cells (HSC), and hepatocytes in an ethanol and lipopolysaccharide (LPS)-induced two-hit model in vitro and in vivo. We determined that siRNA-directed silencing of Akt2, but not Akt1, significantly suppressed cell inflammatory markers in HSC and Kupffer cells. Although both Akt1 and Akt2 inhibited cell proliferation in HSC, only Akt2 inhibited cell migration. Both Akt1 and Akt2, but not Akt3, inhibited fibrogenesis in hepatocytes and HSC. In addition, our in vivo results show that administration of chronic ethanol, binge ethanol and LPS (EBL) in wild-type C57BL/6 mice activated all three Akt isoforms with concomitant increases in activated forms of phosphoinositide dependent kinase-1 (PDK1), mammalian target-of-rapamycin complex 2 (mTORC2), and PI3K, resulting in upregulation in expression of inflammatory, proliferative, and fibrogenic genes. Moreover, pharmacological blocking of Akt2, but not Akt1, inhibited EBL-induced inflammation while blocking of both Akt1 and Akt2 inhibited pro-fibrogenic marker expression and progression of fibrosis. Our findings indicate that Akt isoforms play unique roles in inflammation, cell proliferation, migration, and fibrogenesis during EBL-induced liver injury. Thus, close attention must be paid when targeting all Akt isoforms as a therapeutic intervention.

## 1. Introduction

Chronic alcohol abuse resulting in alcoholic liver disease (ALD) is one of the leading causes of morbidity and mortality worldwide [1]. Long-term excessive alcohol consumption can cause alcoholic steatohepatitis, which is accompanied by steatosis, hepatocyte necrosis, and inflammation [2] that further promotes a wound healing response and the deposition of extracellular matrix (ECM) resulting in fibrosis and eventually cirrhosis. Despite the many advances made in understanding the molecular mechanisms involved in the development of the disease, liver transplantation remains the only treatment for end-stage liver disease [3]. Therefore, there is an urgent need to find novel actionable targets that contribute to the development of inflammation and fibrosis during ALD.

The activities of the different liver cells types including hepatocytes, Kupffer cells, and hepatic stellate cells (HSC) contribute to the progression of ALD. Epidemiological studies further suggest that ethanol alone is not enough to cause advanced ALD and other insults such as lipopolysaccharide (LPS) are necessary for the disease to progress from steatohepatitis to fibrosis and ultimately cirrhosis [4,5]. It is known that LPS is often elevated in alcoholic patients as well as in various animal models of alcoholic liver injury [5,6]. Thus, the well-accepted model of chronic ethanol and LPS-induced fibrosis in mouse liver is referred to as a “two hit” model of ALD [7,8]. In this model, ethanol acts as the first hit and gets oxidized to its toxic metabolite, acetaldehyde in hepatocytes [9], while ethanol-induced leaky gut leads to increased LPS that acts as the second hit to activate nuclear factor kappa B (NFκB) signaling in Kupffer cells and the production of the proinflammatory cytokines [10,11,12]. Together, this leads to the activation of HSC resulting in a pro-fibrogenic cascade promoting fibrosis [13,14,15,16,17,18,19,20].

Akt, also known as protein kinase B, is a serine/threonine protein kinase that plays a central role in all aspects of the development of ALD process, including promoting cell proliferation, migration, and transcription while impairing apoptosis. The Akt family of kinases consists of three distinct isoforms named Akt1, Akt2, and Akt3. Separate genes encode these isoforms. They share a high degree of amino acid homology and are presumably activated by similar signaling pathways in a PI3K- dependent manner [21]. The production of phosphatidylinositol (3,4,5)-triphosphate (PIP3) by PI3K in the plasma membrane leads to Akt activation via phosphorylation by phosphoinositide-dependent kinase-1 (PDK1) on distinct residues on Akt1 (T308, T450), Akt2 (T309), and Akt3 (T305) [22]. The three kinases are also activated by phosphorylation on residues serine S473 for Akt1, S474 for Akt2, and S472 for Akt3 by mammalian target of rapamycin complex 2 (mTORC2) [23,24,25,26].

The Akt kinases have been the subject of extensive investigation and it is generally assumed that they play redundant and overlapping roles. However, a handful of studies suggest the kinases have distinct functions. For example, mice lacking Akt1 display enhanced apoptosis and growth retardation [27], whereas Akt2 knockdown mice display a diabetic phenotype with insulin resistance [28]. In contrast, the role of the brain-enriched isoform, Akt3, remains unclear. Akt1 and Akt2 have also been shown to play opposite roles in regulating migration and invasion in breast cancer [29] while they both inhibit prostate cancer cell migration and invasion [30,31]. At present, very little is known about Akt functions in the liver and with respect to alcoholic liver injury. Thus, the present study was designed to identify the isoform-specific roles of Akt in inflammation and fibrogenesis in ethanol and LPS-induced liver injury.

## 2. Materials and Methods

Animals and Diet: Eight-week-old, wild-type female (females are more susceptible to alcohol-induced liver damage than males) C57BL/6 mice (~25 g body weight) from Charles River, Wilmington, MA were housed in pairs in plastic cages, in a temperature-controlled room at 25 °C with 12 h light–dark cycle. All animals were fed a pelleted commercial diet (Purina Rodent Chow, #500, TMI Nutrition, St. Louis, MO, USA) during the first week of acclimation. Experiments were performed according to the approved institutional animal care and use committee protocol. Mice were randomly divided into 7 groups of 4 rats each, and were pair-fed Lieber DeCarli control or ethanol liquid diets (36% total fat calories) with high-ω3 fatty acids (14.1% of calories as ω3 fatty acids) fish oil for 4 weeks. The diets are isocaloric and their formulations are according to the modified method of Lieber and DeCarli [32] with the recommended normal nutrients, vitamins, and minerals according to the AIN-93 diet. Thus, 36% of the total energy of ethanol diet is from fat, 20% from protein, 36% from ethanol, and the rest from carbohydrate. The corresponding isocaloric control diet has isoenergetic amounts of dextrin-maltose in place of ethanol. Ethanol concentrations in the liquid diet were gradually increased starting at 1% on day 1, reaching 5% over a seven-day period to allow the animals to adapt. The mice fed with ethanol diets were also administered a single dose of 5 g/kg body weight of ethanol by gavage in the absence or presence of LPS (2 mg/kg body weight, i.p.) 6 h prior to euthanizing the animal. This two-hits model of ethanol binge and LPS (EBL)-induced liver injury was adapted from the two-hit ethanol/LPS model described by Hoek et al. [8] and ethanol/binge model by Bertola et al. [7]. The selective Akt1 inhibitor, A674563 (Selleckchem, Houston, TX, USA) (20 mg/kg i.p) was dissolved in 5% dextrose, the Akt2 inhibitor, CCT128930 (Selleckchem) (50 mg/kg i.p) was dissolved in 10% DMSO, 5% Tween 20, and 85% saline. They were administered as daily doses for 1 week prior to LPS administration [33,34,35,36]. Efficiency of Akt1 and Akt2 inhibition in a EBL in vivo mouse model is shown in supplementary Figure S2.

Cell Culture. Primary human HSC were isolated as described by collagenase and protease digestion and fractionation on an Optiprep (Sigma, Saint Louis, MO, USA) gradient from human liver biopsies of patients with morbid obesity by an approved protocol (IRB 070701). Activated HSC were grown to confluence in Dulbecco’s modified Eagle’s medium (Gibco; Invitrogen, Grand Island, NY, USA), supplemented with 10% (*v*/*v*) fetal bovine serum (FBS), 1% (*v*/*v*) amino acids (Cellgro, Herndon, VA, USA), and 1% (*v*/*v*) penicillin/streptomycin (Gibco; Invitrogen). HSC were treated with 200 μM of acetaldehyde and/or 1 μg/mL of LPS for 3 h and maintained at 37 °C in a 5% CO_2_ humidifier incubator. Human Kupffer Cells that express CYP2E1 were obtained from Life Technologies, Grand Island, NY (# HUKCCS, USA). VL17A hepatocytes were provided by Dr. Dahn L Clemens from the Liver Study Unit, VA Medical Center (Omaha, Nebraska, USA). VL17A cells (HepG2 cells that constitutively express both cytochrome P450 2E1 (CYP2E1) and alcohol dehydrogenase (ADH) were generated as described [37]. Kupffer Cells and VL17A hepatocytes were grown to confluence in Dulbecco’s modified Eagle’s medium F12, from ATCC (Manassas, VA, USA) supplemented with 10% (*v*/*v*) fetal bovine serum (FBS), 1% (*v*/*v*) amino acids (Cellgro, Herndon, VA, USA), and 1% (*v*/*v*) penicillin/streptomycin. (Gibco; Invitrogen). VL17A hepatocytes were also supplemented with 5 mg/L insulin-transferrin-sodium selenite (ITS, Roche, Branchburg, NJ, USA) and dexamethasone 10^−7^ M (Sigma). Geneticin and Zeocin (400 mg/l; Life Technologies, Grand Island, NY, USA) were added to the culture media as selective antibiotics. Kupffer cells and VL17A hepatocytes were exposed to 100 mM ethanol for 72 h [11,12,38]. Cells were also treated with 100 μg/mL of LPS [39] for 3 h, in the absence or presence of acetaldehyde or ethanol. Cultures were maintained in fetal bovine serum-containing medium until 16 h before start of the experiments. They were washed with phosphate buffered saline (PBS) and the medium was replaced with serum-free medium containing 0.1% fetal bovine serum. Cells were treated and maintained at 37 °C in a 5% CO_2_ humidifier incubator.

Akt1 and Akt2 siRNA Transfection: The individual Akts were selectively silenced in HSC, Kupffer cells, and VL17A cell cultures by transfecting with 50 nM of the respective siGENOME SMART pool human Akt1 or Akt2 and siControl: Non-targeting siRNA pool (GE Healthcare life and science, Pittsburg, PA, M-003000, M-003001, and D-001206, respectively) using DharmaFECT1 transfection reagent (Thermo Scientific, T-2001-03) according to the manufacturer’s instructions. After verifying knock-down of each Akt isoform by using Western Blot (transfection efficiency >90%), the respective specific Akt-isoform silenced HSC were exposed to clinically relevant concentrations of acetaldehyde (200 µM) for 24 h [40], whereas silenced Kupffer cells and VL17A hepatocytes were exposed to 100 mM ethanol for 72 h [11,12,38]. Sealed parafilm was used to minimize evaporation of the volatile acetaldehyde and ethanol. Cells were also treated with 100 μg/mL of LPS [39] for 3 h, in the absence or presence of acetaldehyde or ethanol. Efficiency of Akt1, Akt2, and Akt3 transfection is shown in supplementary Figure S2.

AST and ALT Measurement: Plasma alanine aminotransferase (ALT) and aspartate aminotransferase (AST) were measured using commercial kits according to the manufacturer’s instructions (Teco Diagnostics, Anaheim, CA, USA).

Total, Nuclear, and Cytosolic Protein Extraction: Total protein was extracted from liver or cultured cells by homogenization in lysis buffer containing 1 mol/L Tris (pH 8), 5 mol/L NaCl, 0.5 mol/L EDTA, 0.5 mol/L NaF, 100 mmol/L sodium pyrophosphate, 100 mmol/L Na_3_VO_4_ and, 200 mmol/L phenylmethylsulfonyl fluoride. Nuclear and cytosolic fractions were isolated using a commercial kit (Thermo Scientific, Rockford, IL, USA).

SDS-PAGE and Western Blot Analysis: Protein concentrations were determined using the bicinchoninic acid assay (Pierce Chemical Rockford, IL. USA). For Western Blots, antibodies against p-PI3K, p-PDK1, p-mTOR, NFκB-p65, IκB, and PDGFβR were purchased from Cell Signaling (Danvers, MA, USA); antibodies against Akt1, Akt2, Akt3, p-IκB, αSMA, fibronectin, and lamin B1 were obtained from Abcam (Cambridge, MA, USA); and antibodies against collagen1α2 and β-actin were purchased from Santa Cruz Biotechnologies (Santa Cruz, CA, USA) and Aldrich-Sigma, respectively. Proteins were detected with a NEN Life Science Products Renaissance enhanced chemiluminescence system (PerkinElmer, Waltham, MA, USA) according to the manufacturer’s recommendations.

RNA Extraction and Quantitative RT-PCR: RNA from liver tissue or cultured cells was extracted using TriZol reagent (Life Technologies, Carlsbad, CA, USA). cDNA templates were synthesized and quantitative RT-PCR was performed as previously described [41]. 40S ribosomal protein *Gapdh* was used as the standard gene. Ratios of the target gene and *Gapdh* gene expression levels were calculated by subtracting the threshold cycle number (C_t_) of the target gene from the C_t_ of 40S ribosomal protein *Gapdh* and raising to the power of the negative of this difference. Target gene expression is expressed relative to 40S ribosomal protein *Gapdh* gene expression.

Hydroxyproline Assay: Hydroxyproline content in liver tissue was measured colorimetrically using a commercial kit (Sigma).

MTT Proliferation Assay: HSC proliferation was assessed using the 3-(4,5-dimethylthiazol-2-yl)-2,5-diphenyl tetrazolium bromide (MTT) assay. Cells were plated in 96-well tissue culture plates at a concentration of 3000 cells/well. After 24 h of quiescence, the cells were cultured for 24 h or 48 h with media containing 0.1% fetal bovine serum. At the end of the treatment, 20 μL MTT solution (5 mg/mL in PBS) was added to each well and incubated for an additional 2 h at 37 °C. The colored formazan product was then dissolved in 150 μL of MTT solvent (4 mmol/L HCl and 0.1% Nonidet P-40 in isopropanol) and detected in a plate reader at 570 nm absorbance.

Migration Assay: Cell migration was measured in a scratch-wound assay. The human HSC were grown to confluence and were then serum-deprived for 24 h. After the medium was removed, a scratch wound was inflicted in a straight line with a pipette tip. The plates were then rinsed with PBS and incubated with Dulbecco’s modified Eagle’s medium supplemented with acetaldehyde and/or LPS. Wound closure was visualized and photographed after 24 h using a light microscope. Images were analyzed using Adobe Photoshop CS (Adobe Systems Inc., San Jose, CA, USA). The gap distances between the gap of migrating HSC were measured.

Statistical Analysis: All experiments were performed in triplicate and data are expressed as mean ± SE. Statistical differences between experimental groups were analyzed by the Student’s t-test and *p* ≤ 0.05 was considered significant.

## 3. Results

### 3.1. Ethanol and LPS Induce Liver Injury and Activate Akt Signaling Pathways

**Figure 1:**
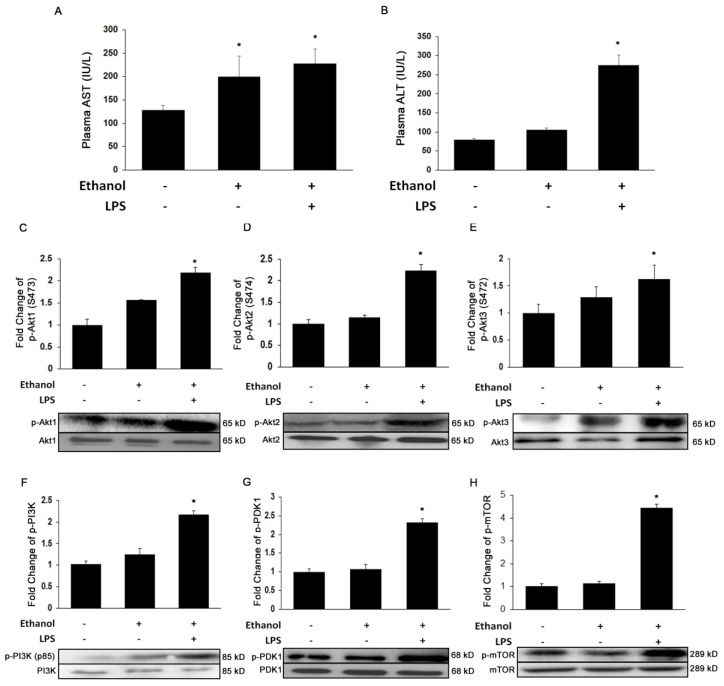
Ethanol and lipopolysaccharide (LPS) induces liver injury and activates Akt signaling pathway. Biochemical analysis of plasma (**A**) aminotransferase (AST) and (**B**) alanine aminotransferase (ALT); and Western Blot analysis of (**C**) p-Akt1, (**D**) p-Akt2, (**E**) p-Akt3, (**F**) p-PI3K, (**G**) p-PDK1, and (**H**) p-mTOR. Protein was extracted from whole livers of controls and mice treated with ethanol binge (EB) and ethanol binge and LPS (EBL) (*n* = 6). All values are means of quadruplicate experiments ± SE after correcting for the expression of β-actin. * *p* ≤ 0.05 as compared to control.

To determine whether ethanol and LPS effectively induce significant liver injury, we measured the injury markers, AST and ALT. As shown in Figure 1A, ethanol alone or with LPS significantly increased plasma AST levels by 1.6-fold and 1.8-fold (*p* ≤ 0.05), respectively. Similarly, plasma ALT levels were also markedly increased by 1.2-fold by ethanol alone, and to an even higher extent with added LPS (3.4-fold, *p* ≤ 0.05) (Figure 1B). To further investigate if ethanol and LPS-induced liver injury results in Akt activation, we evaluated protein phosphorylation of the three Akt isoforms and the phosphorylation status of the kinases that activate Akt. Ethanol alone or in combination with LPS significantly increased the expression of all three Akt isoforms by ~2-fold (Figure 1C,E) accompanied by corresponding increases in the phosphorylation of PI3K, PDK1, and mTOR by 2-, 2.5-, and 4-fold (*p* ≤ 0.05), respectively (Figure 1F,H).

### 3.2. Akt2, But Not Akt1, Is Involved in Ethanol and LPS-Induced Nuclear Translocation of NFκB-p65 and Induction of Pro-Inflammatory Cytokines In Vitro and In Vivo

**Figure 2:**
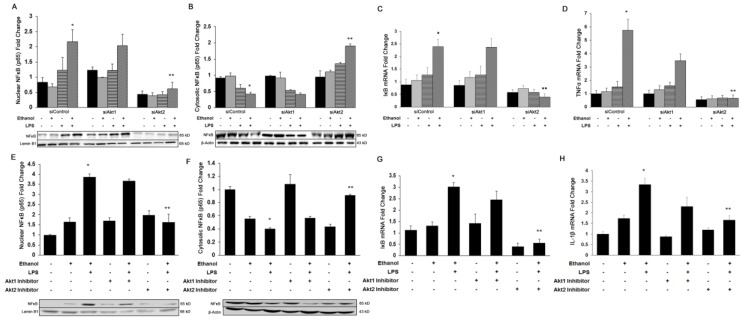
Role of Akt isoforms on ethanol and LPS-induced nuclear translocation of NFκB-p65 and induction of pro-inflammatory cytokines in vitro and in vivo. Nuclear or cytosolic protein and total RNA was extracted from human Kupffer cells transfected with siAkt1 or siAkt2 and treated with ethanol, LPS, or both for Western blot analysis of (**A**) nuclear NFκB-p65, (**B**) cytosolic NFκB-p65, and qPCR analysis of (**C**) *IκB*, and (**D**) *Tnfα*. Nuclear or cytosolic protein and total RNA was extracted from mouse liver tissue treated with Akt1 or Akt2 inhibitors and treated with EB and EBL (*n* = 6) for Western blot analysis of (**E**) nuclear NFκB-p65, (**F**) cytosolic NFκB-p65, and qPCR analysis of (**G**) *IκB* and (**H**) *Il1β*. All values are means of triplicate experiments ± SE after correcting for the expression of Lamin B (nuclear), β-actin (cytosolic), or glyceraldehyde-3-phosphate dehydrogenase (*Gapdh*) (qPCR). * *p* ≤ 0.05 as compared to control, ** *p* ≤ 0.05 as compared to ethanol Ethanol, LPS, or both, and/or LPS or EBL.

It is well known that ethanol and LPS-mediated activation of Akt signaling plays a crucial role in the onset of inflammation [42]. To identify the Akt isoform-specific role in the induction of an inflammatory response during liver injury, we used both in vivo inhibition and in vitro silencing of Akt1 and Akt2 isoforms in Kupffer cells. Because silencing of Akt3 did not alter ethanol and LPS- mediated liver damage, we did not include these data in this study. In cultured Kupffer cells, added ethanol and LPS increased the nuclear translocation of NFκB-p65 by 2.4-fold (*p* ≤ 0.05) (Figure 2A) with a reciprocal 50% decrease in cytosolic NFκB-p65 (*p* ≤ 0.05) (Figure 2B). These changes were associated with a significant increase in the gene expression of IκB by 2.4-fold (*p* ≤ 0.05) (Figure 2C), and resulted in the up-regulation of TNFα mRNA levels by 5.5-fold (*p* ≤ 0.05) (Figure 2D). Importantly, silencing of only the Akt2 isoform significantly decreased the expression of *Iκb* gene expression, and significantly inhibited NFκB-p65 activation by 80%, resulting in the down-regulation of TNFα mRNA by 90% (*p* ≤ 0.05). Similarly, in the in vivo mouse model of ethanol and LPS-induced liver injury, ethanol alone or in combination with LPS significantly increased nuclear translocation of NFκB-p65 by 2-fold and 3-fold (*p* ≤ 0.05), respectively (Figure 2D). Also, as seen in vitro, treatment with only the Akt2 inhibitor decreased nuclear translocation of NFκB-p65 by 50% (*p* ≤ 0.05) (Figure 2D). Ethanol alone or with LPS also significantly up-regulated gene expression of the pro-inflammatory cytokine, IL-1β by 1.7-fold (*p* ≤ 0.05) and 3.3-fold (*p* ≤ 0.05). Only mice treated with the Akt2 inhibitor showed a marked down-regulation of IL-1β mRNA by 50% (*p* ≤ 0.05) in mice additionally treated with ethanol and LPS (Figure 2E). These results indicate that only Akt2 plays a critical role in regulating the inflammatory process during ALD.

### 3.3. Both Akt1 and Akt2 Are Involved in Ethanol and LPS Induced Fibrosis

**Figure 3:**
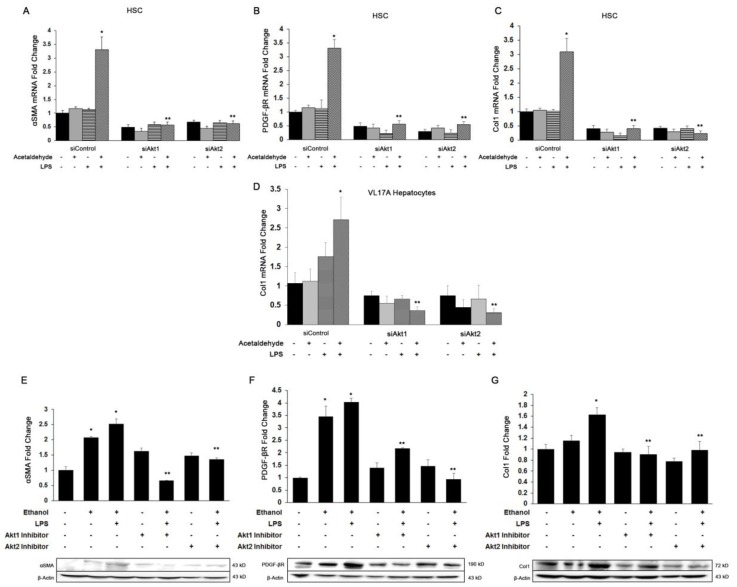
Role of Akt1 and Akt2 in ethanol and LPS-induced fibrogenesis. Total RNA was extracted from human hepatic stellate cells (HSC) or VL17A hepatocytes transfected with siAkt1 or siAkt2 and treated with acetaldehyde/ethanol, LPS, or both for quantitative PCR analysis of (**A**) *αS*ma, (**B**) *Pdgfβ* receptor, (**C**) *Col1α1*, and (**D**) *Col1α1* from VL17A cells. Total protein was extracted from whole liver tissue from mice of various groups (*n* = 6) to determine the protein expression of (**E**) αSMA, (**F**) PDGFβ Receptor, (**G**) Col1 by Western blot analysis. All values are means of triplicate experiments ± SE after correcting for the expression of glyceraldehyde-3-phosphate dehydrogenase (*Gapdh*) (mRNA) or β-actin (protein). * *p* < 0.05 as compared to control; ** *p* < 0.05 as compared to acetaldehyde + LPS or EBL.

**Figure 4:**
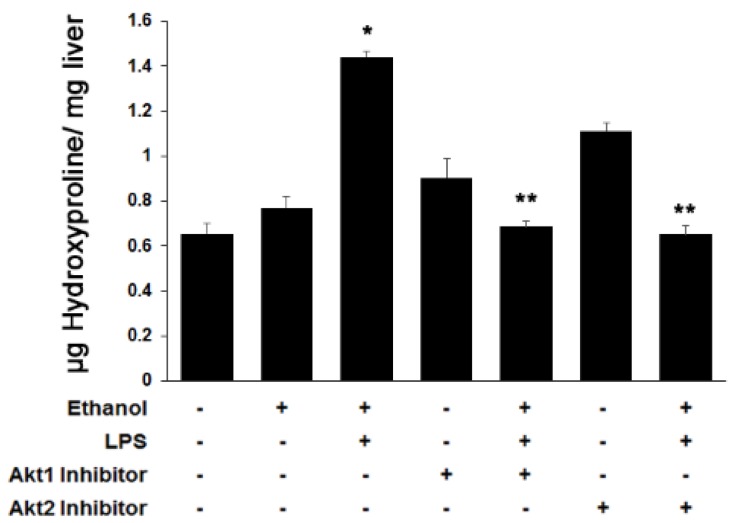
Role of Akt1 and Akt2 in ethanol and LPS-induced fibrosis. Liver tissue from various groups was used to determine hydroxyproline content (indicative of collagen fibers) to determine the extent of fibrosis. * *p* < 0.05 as compared to control, **p < 0.05 as compared to EBL.

Acetaldehyde generated from ethanol oxidation along with the secretion of several growth factors and pro-inflammatory cytokines by Kupffer cells can lead to fibrosis via trans-differentiation of HSC into myofibroblasts [43]. Therefore, we determined the effect of Akt isoform-specific silencing on ethanol and LPS-mediated fibrogenesis. Because HSC display negligible alcohol dehydrogenase activity, we added physiologically relevant concentrations of acetaldehyde (the immediate metabolic product of ethanol oxidation) to the HSC cultures. As shown in Figure 3A–C, acetaldehyde and LPS up-regulated the expression of smooth muscle actin (*αSma*), platelet-derived growth factor receptor (*Pdgfβr*), and collagen1 (*Col1α1*) mRNA by 2.5-, 3-, and 4-fold, respectively, in HSC cultures. Knockdown of either Akt1 or Akt2 significantly inhibited the acetaldehyde and LPS-mediated effect on expression of αSMA by 50% and 80%, respectively, PDGFβR by 90%, and Col1α1 by 90% and 95%, respectively. Since hepatocytes also contribute to collagen production in early fibrosis, we measured *Col1α1* mRNA in VL17A hepatocytes. Similarly, ethanol and LPS induced expression of *Col1α1* mRNA in VL17A hepatocytes by 3.4-fold, and knockdown of Akt1 or Akt2 significantly inhibited the ethanol and LPS effect by about 80% (Figure 3D). Consistent with the in vitro findings, ethanol alone or in combination with LPS in mice significantly induced the protein expression of the pro-fibrogenic markers by 2- to 4-fold (*p* ≤ 0.05) (Figure 3E–G). Treatment with either the Akt1 or Akt2 inhibitor led to a significant decrease in the expression of αSMA by 74% or 46% (*p* ≤ 0.05), PDGFβR by 46% or 76% (*p* ≤ 0.05), and Col1 by 45% or 40% (*p* ≤ 0.05), respectively. Furthermore, as shown in Figure 4, hydroxyproline, another marker of fibrosis, was increased 1.3-fold (*p* ≤ 0.05) in animals treated with ethanol alone or up to 2.3-fold (*p* ≤ 0.05) with added LPS (*p* ≤ 0.05). Treatment with either the Akt1 or Akt2 inhibitor completely blocked this effect. Akt1 or Akt2 inhibitors prevented the ethanol and LPS-induced effect, thus reducing the extent of fibrosis. Together, these results indicate that both Akt1 and Akt2 are involved in ethanol and LPS-mediated hepatic fibrosis. 

### 3.4. Akt1 and Akt2 Are Involved in HSC Proliferation, but Only Akt2 Regulates Migration

**Figure 5:**
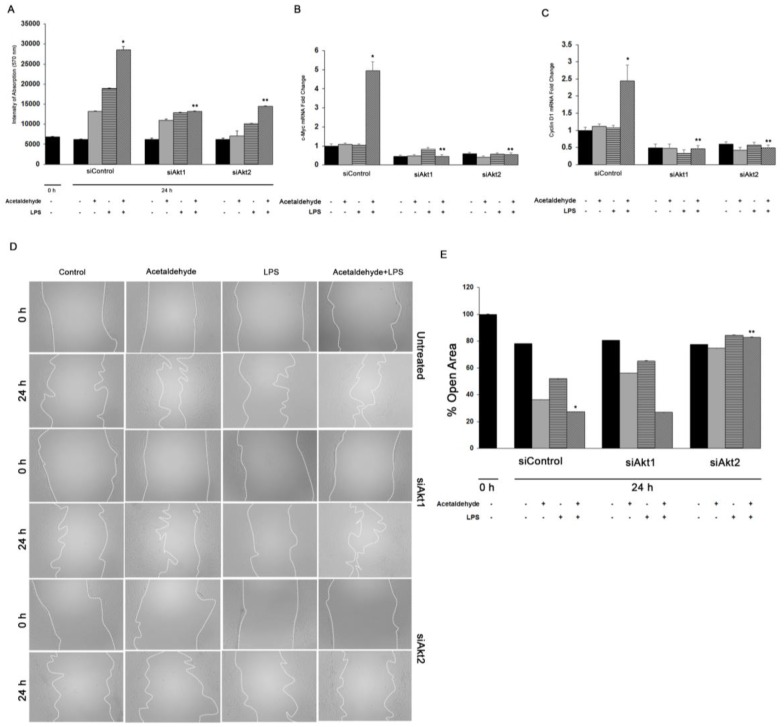
Role of Akt1 and Akt2 in HSC proliferation and migration. (**A**) Cell proliferation was performed by MTT assay of human HSC transfected with siAkt1 or siAkt2 and treated for 3 h with acetaldehyde, LPS, or both. Quantitative qPCR analysis of (**B**) *c-Myc* and (**C**) *Cyclin D1* was performed with total RNA obtained from human HSCs. All values are means of triplicate experiments ± SE after correcting for the expression of *S18*. (**D**) Cell migration was determined after wounding a confluent culture and measuring the gap area at the time points indicated in the figure. Panel E shows percent of open area. Values are means of triplicate experiments ± S.E. * *p* < 0.05 as compared to control, ** *p* < 0.05 as compared to acetaldehyde and LPS.

We have previously shown that the expression of PDGFβR and the activation of Akt signaling lead to HSC proliferation [44]. Therefore, we examined the effect of Akt1 or Akt2 silencing on acetaldehyde and LPS-mediated HSC proliferation. As shown in Figure 5A, acetaldehyde and LPS alone induced a 2-fold or 2.5-fold increase, respectively, in HSC proliferation. The combination of acetaldehyde and LPS induced an even greater increase in HSC proliferation by up to 3.5-fold. Knockdown of either Akt1 or Akt2 inhibited proliferation by ~55% (Figure 5A). Additionally, the combination of acetaldehyde and LPS significantly up-regulated expression of the cell cycle regulating genes, *cMyc* and *cyclin D1*, by 5-fold and 2.5-fold, respectively. Akt1 or Akt2 silencing markedly downregulated the mRNA levels of *cMyc* and *Cyclin D1* by ~90% (Figure 5B,C).

As shown in Figure 5D,E, acetaldehyde and LPS also induced HSC migration resulting in wound closure after 24 h by 63% and 48% respectively, compared to control with closure of only 22%. Acetaldehyde and LPS treatment led to a further increase in migration with 73% of the wound closed at 24 h. Silencing of Akt1 did not alter HSC migration when compared to cells treated with the scrambled siRNA. In contrast, there was a significant decrease in HSC migration in cells silenced for Akt2, to only 17% of that observed in cells treated with both acetaldehyde and LPS. Together, these results reflect the functional specificity between of Akt1 and Akt2 in mediating HSC proliferation and migration.

## 4. Discussion

To our knowledge, this is the first study to examine the specific roles of the Akt isoforms in regulating chronic alcohol-mediated pathogenesis of ALD. While both Akt1 and Akt2 isoforms were found to regulate fibrogenesis and proliferation in hepatocytes and HSC, only Akt2 differentially regulated inflammation and migration. Interestingly, Akt3 was not found to play a specific role in these cellular processes.

Although the three Akt isoforms share a high degree of structural homology (81% amino acid identity with Akt2 and 83% with Akt3) [21], accumulating evidence suggests that Akt1 and Akt2 are ubiquitously expressed [45], whereas, Akt3 is enriched in the brain and testes and that each isoform is differentially implicated in the pathogenesis of various diseases [46,47,48,49].

Our results showed that Akt3 knockout does not alter ethanol and LPS-induced inflammation or fibrosis. However, ethanol and LPS do induce the expression of Akt3 in the liver, suggesting that this isoform may play a redundant or presently unidentified role in the two-hit model of alcohol-induced hepatic inflammation and fibrosis [48,49].

During alcoholic liver injury, LPS stimulates Kupffer cells, via Toll-like receptor 4 binding and activation of the Akt/NFκB signaling pathway leading to the generation of pro-inflammatory cytokines [13,14]. More recently, the specific involvement of Akt1 and Akt2 in eliciting an inflammatory response in various tissues and diseases has been reported. For example, Di Lorenzo et al. have demonstrated that Akt1 is critical for acute inflammation and histamine-mediated vascular leakage [50]. On the other hand, Arranz et al. found that Akt2, but not Akt1, ablation protected against dextran sulfate sodium-induced inflammatory bowel disease in mice [51]. Similarly, only Akt2 deficiency attenuated bleomycin-induced pulmonary inflammation [52]. Our results show that ethanol and LPS-induced activation of IκB, NFκB translocation to the nucleus, and the consequent production of the pro-inflammatory cytokines: TNFα, IL1β, in Kupffer cell cultures, and in the EBL mouse model. Deletion of only Akt2 protected against ethanol and LPS-induced inflammatory response, suggesting that only this isoform plays a critical role in the development of liver inflammation associated with ALD.

Ethanol and LPS and/or acetaldehyde and LPS also upregulated the fibrogenic markers, PDGFβR and αSMA, and collagen. Blocking of both Akt1 and Akt2 reversed the effect, both in culture and in the EBL mouse model, suggesting that both isoforms play an active role in fibrogenesis. This is consistent with results from Bibaki et al., which demonstrated that miR-29a, miR-185, and their targets Akt1 and Akt2, were involved in the development of idiopathic pulmonary fibrosis and lung cancer [53]. However, Lan et al. showed that only Akt2 was involved in renal fibrosis [54]. In contrast, Ock et al. showed that neither cardiac fibrosis nor apoptosis was detected in Akt1^−/−^/Akt2 knockout hearts [55]. Thus, the participation of Akt1 and/or Akt2 in the pathogenesis of fibrosis likely varies among cell types and pathogenic states. 

Proliferation and migration of HSC during hepatic injury is essential for wound healing and fibrosis of the liver [44,56]. Our results also revealed that acetaldehyde and LPS administration caused a significant increase in proliferation and migration of HSC. Silencing of Akt1 and Akt2 decreased acetaldehyde and LPS-mediated proliferation and decreased mRNA and protein expression of cell cycle regulating genes. However, only knockdown of Akt2 inhibited HSC migration. In contrast, Virtakoivu et al. showed that both Akt1 and Akt2 negatively regulated prostate cancer migration and invasion [30]. Thus, it is evident that Akt isoforms exhibit functional redundancy as expected, but also have non-redundant physiological activities that vary among tissue types.

Taken together, these data indicate that Akt family members differentially regulate specific functions during ALD, in vivo and in vitro. These results imply that differential expression, activation, or localization of the isoforms may play dominant roles in determining isoform-specific functions. However, differences in enzyme activity and/or substrate specificity may also contribute to the differential effects of Akt isoforms on regulating a wide range of cellular processes, including inflammation, fibrosis cell proliferation, and migration. It is important to note that the basis of isoform-specific signaling is likely to vary depending on the type of cell or tissue. Indeed, recent studies suggest that the substrates targeted by different AKT isoforms can vary depending on cellular and tissue context [57].

While the clinical ramifications of acute and chronic ethanol exposure are well known, the current explanations of cellular mechanisms leading to tissue damage and alcohol-related gene expression patterns lag. Our present study suggests that each Akt isoform plays a unique role in the development of inflammation, cell proliferation, migration, and fibrogenesis during alcoholic liver injury. Our study reveals a better understanding of the basic pathophysiology of the signaling pathways associated with ALD progression and may lead to designing selective new therapies against actionable targets to treat alcoholic patients.

## Supplementary Materials

The following are available online at https://www.mdpi.com/2073-4409/8/11/1337/s1, Figure S1: Efficiency of Akt1 and Akt2 transfection, Figure S2: Efficiency of Akt1 and Akt2 inhibition in a EBL in vivo mouse model.
