# Supplementary material for: Akt1 and Akt2 Isoforms Play Distinct Roles in Regulating the Development of Inflammation and Fibrosis Associated with Alcoholic Liver Disease
Source: Cells. 2019 Oct 29;8(11):1337. doi: 10.3390/cells8111337 (PMC6912497; doi:10.3390/cells8111337)

Supplementary Figure S1. Efficiency of Akt1 and Akt2 transfection. Akt1 and Akt2 silencing in human (A,B) HSC, (B,C) Kupffer cells, and (D,E) VL17A hepatocytes was performed by transfecting with the respective siGENOME SMART pool human Akt1 or Akt2 and siControl: non-targeting siRNA pool (GE Healthcare: M-003000, M-003001, M-003002, and D-001206, respectively) using DharmaFECT1 transfection reagent (Thermo Scientific, T-2001-03). After verifying the knock-down of each Akt isoform, the respective Akt-isoform silenced HSC, Kupffer cells and/or VL17A hepatocytes were exposed to 200 of Acetaldehyde for 24h or 100 mM ethanol for 72h and then LPS (1 µg/ml) was added and incubated for 3 h. to acetaldehyde and/or ethanol and/or LPS.

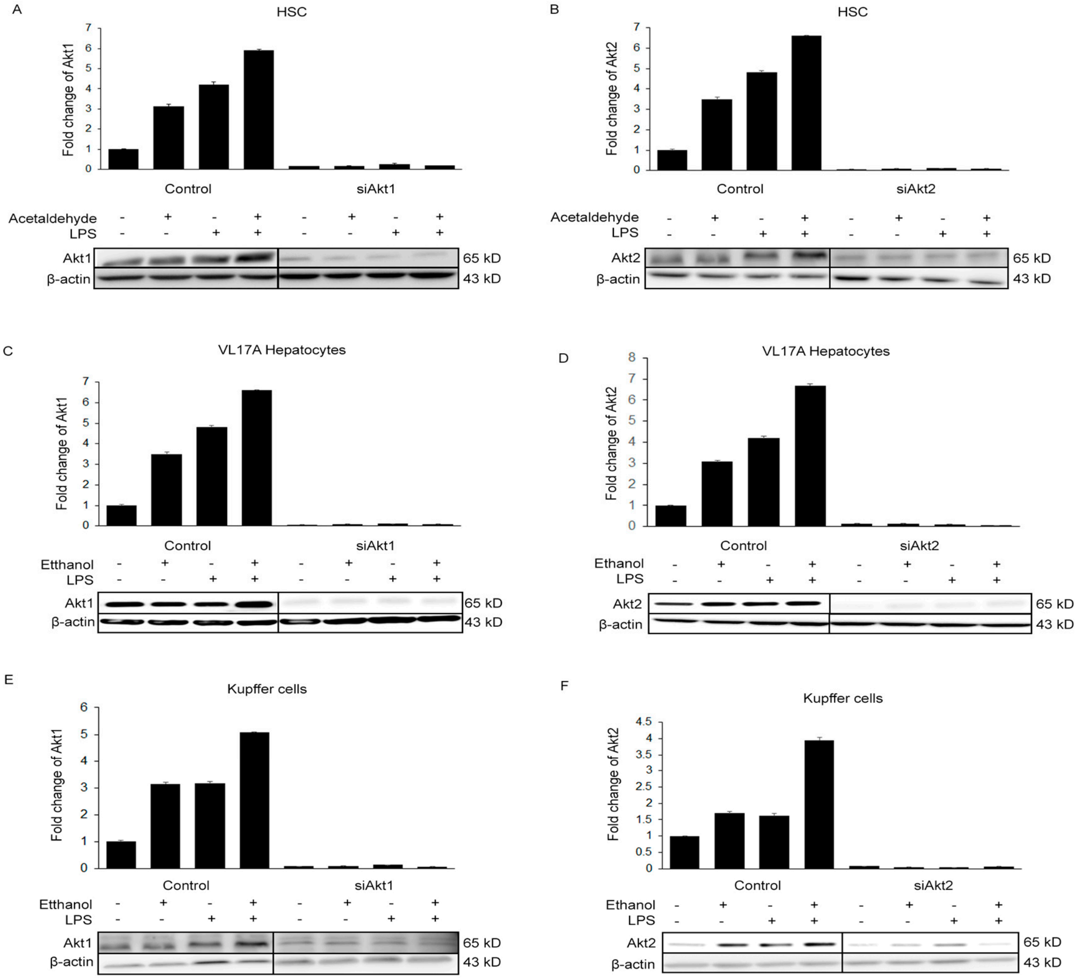

Supplementary Figure S2. Efficiency of Akt1 and Akt2 inhibition in a EBL in vivo mouse model. (A) Akt 1 and (B) Akt2 inhibition in a EBL mouse model was performed by using Akt1 inhibitor (A674563), 20 mg/kg i.p, dissolved in 5% dextrose; and Akt2 inhibitor, (CCT128930) (50 mg/kg i.p) dissolved in 10% DMSO, 5% Tween 20, and 85% saline. Inhibitors were administered as daily doses for 1 week prior to LPS administration.

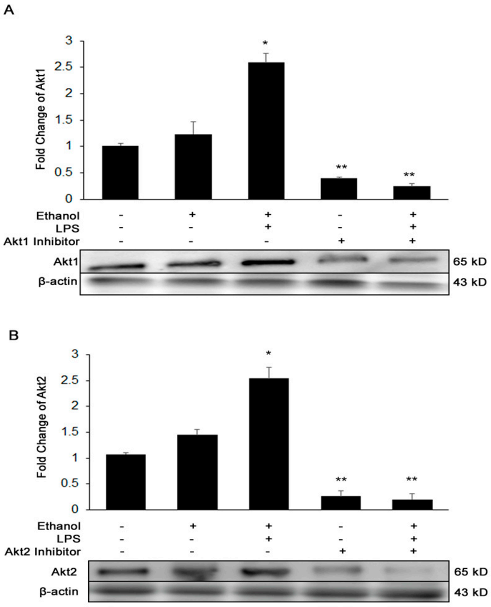

Supplement: Supplementary file 1 [file cells-08-01337-s001.pdf]
